# Treatment of Dry Eye With Intracanalicular Injection of Hydroxybutyl Chitosan: A Prospective Randomized Clinical Trial

**DOI:** 10.3389/fmed.2021.769448

**Published:** 2021-11-18

**Authors:** Tong Lin, Wushuang Wang, Yang Lu, Lan Gong

**Affiliations:** ^1^Department of Ophthalmology, Eye, Ear, Nose, and Throat Hospital of Fudan University, Shanghai, China; ^2^NHC Key Laboratory of Myopia (Fudan University), Laboratory of Myopia, Chinese Academy of Medical Sciences, Shanghai, China; ^3^Shanghai Key Laboratory of Visual Impairment and Restoration, Fudan University, Shanghai, China

**Keywords:** dry eye disease, hydroxybutyl chitosan, intracanalicular occlusion, ocular surface, treatment

## Abstract

**Background:** Punctal/intracanalicular plugs on the market nowadays are all designed before clinical use in treating dry eye disease (DED). To provide an individualized lacrimal drainage system occlusion method and reduce the complications, we developed a “liquid plug” strategy by intracanalicular injection of hydroxybutyl chitosan (HBC) solution, a thermosensitive, phase-changing biomaterial. This study evaluated the efficacy and safety of the HBC plug in treating dry eye disease by comparing it with the VisiPlug absorbable intracanalicular plug.

**Methods:** A monocenter, randomized, controlled clinical trial was performed. Fifty patients with DED were randomized 1:1 to undergo either the HBC injection treatment or the VisiPlug treatment. Ocular Surface Disease Index (OSDI) questionnaire, tear break-up time (TBUT), corneal fluorescence staining (CFS), tear meniscus height (TMH), and phenol red thread test were evaluated at Day 0 (baseline, before treatment) and Weeks 1, 4, and 12.

**Results:** The two groups had a balanced baseline of age, gender, and DED-related characteristics. Both occlusion methods could relieve the symptoms and signs of DED. Significant improvement was found in OSDI, phenol red thread test, and tear meniscus height (*P* < 0.05 compared to baseline) but not in corneal fluorescence staining and tear break-up time (*P* > 0.05). There is no statistically significant difference between HBC injection and VisiPlug at Weeks 1 and 4 (*P* > 0.05). However, at week 12, the HBC injection was not as effective as the VisiPlug in maintaining phenol red thread test (HBC: 5.35 ± 3.22 mm, VisiPlug: 8.59 ± 4.35 mm, *P* = 0.009) and tear meniscus height (HBC: 206.9 ± 47.95 μm, VisiPlug: 242.59 ± 60.30 μm, *P* = 0.041). The numbers of ocular adverse events were relatively low in both groups.

**Conclusions:** The HBC injection showed similar efficacy and safety compared to VisiPlug. The intracanalicular injection of HBC solution proves to be a promising, individualizing method to treat DED.

**Clinical Trial Registration:** This study is registered with the Chinese Clinical Trial Registry (https://www.chictr.org.cn/enindex.aspx), Identifier: ChiCTR1800016603.

## Introduction

Dry eye disease (DED) is a multifactorial disease of the ocular surface characterized by a loss of homeostasis of the tear film and accompanied by ocular symptoms, in which tear film instability and hyperosmolarity, ocular surface inflammation and damage, and neurosensory abnormalities play etiological roles (1). DED can greatly affect people's life and worse symptoms of DED are associated with decreased work productivity levels (2). About 21% of the adults in China are suffering from DED, which causes a great burden to society (3). Management and therapy of DED include treatments for tear insufficiency, tear conservation approaches, treatments for lid abnormalities, anti-inflammation therapy, and others (4). A sequence of treatments is often recommended according to the stage of the disease. In moderate or severe DED cases where tear replacement approaches alone are not enough, lacrimal drainage system occlusion is regarded as a simple and effective tear conservation method (5).

Lacrimal drainage system occlusion is commonly undertaken using punctal/intracanalicular plugs, including absorbable and non-absorbable plugs. Though featuring numerous materials (6–9), plugs available on the market currently are all shaped into a certain design before clinical use and are difficult to apply to individual treatment. Notably, the most common complication of punctal occlusion, spontaneous plug extrusion, is often caused by undersized plugs, which could lead to decreased efficacy and economic losses to DED patients (10). Thus, an individualized design of plug is necessary for better efficacy as well as fewer complications.

Recently, we have designed a novel type of “liquid plugs” using hydroxybutyl chitosan (HBC), a thermosensitive and dissolving material with good biocompatibility (11). When injected into the canaliculus, the HBC solution instantly formed a hydrogel plug at the body temperature and turned into an absorbable intracanalicular HBC plug. With the thermosensitive phase-changing feature, the HBC plug can fit all kinds of canaliculi. In our previous study, it was effective for treating the rabbit DED model (12). By comparing to the VisiPlug absorbable intracanalicular plug, this clinical study was undertaken to confirm the efficacy and safety of HBC plug and also to explore the difference between the HBC plug and other traditional absorbable punctal plug in treating DED.

## Methods

### Study Design

A monocenter, randomized, controlled clinical trial was performed to evaluate the difference of safety and efficacy between the intracanalicular injection of HBC solution (Qisheng Biologic Agent Limited Company in Shanghai, China) and the absorbable intracanalicular plug, VisiPlug (Lacrimedics, Inc., United States), in DED patients. The study was performed in the Eye & ENT Hospital of Fudan University, was registered in the Chinese Clinical Trial Registry (Identifier: ChiCTR1800016603), was conducted in compliance with the Declaration of Helsinki, and was approved by the ethical committee of Eye & ENT Hospital of Fudan University. Written informed consent was obtained from all participants.

**Figure 1:**
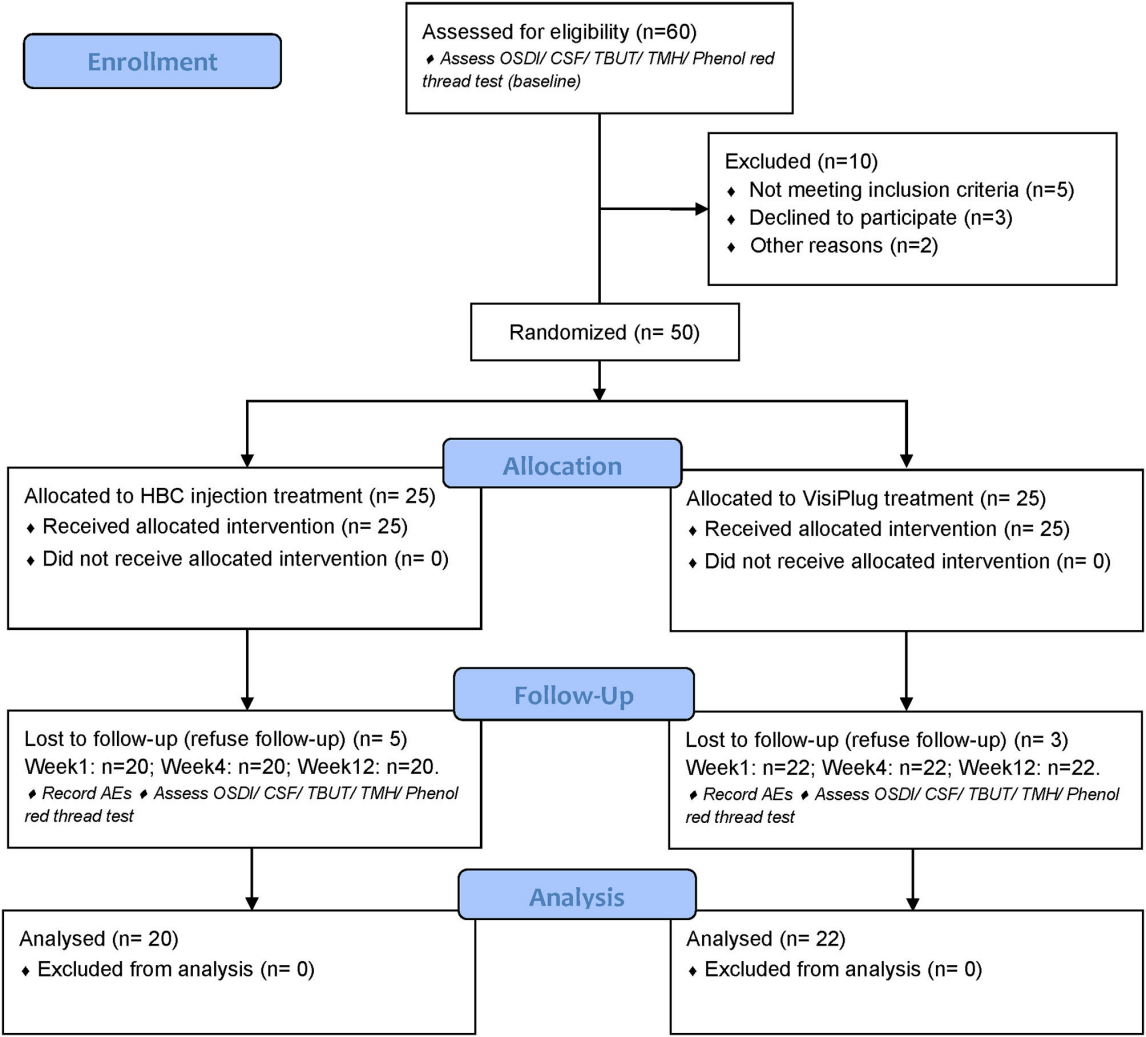
CONSORT flow diagram of the study.

The clinical trial consisted of the intracanalicular occlusion treatment as well as a 12-week visit after the treatment (Figure 1). After informed consent was obtained, DED patients who met all criteria began the study and were randomized into either HBC plug treatment group or VisiPlug treatment group in a 1:1 ratio. In the HBC group, the HBC solution was injected into the puncta of the upper and lower canaliculi in both eyes, respectively. In the VisiPlug group, the plugs were placed into the puncta of the upper and lower canaliculi in both eyes. Patients could maintain the artificial tears therapy as before. Patients attended a total of 5 study visits: visit 0, day−14, screening; visit 1, day 0, randomization and treatment (baseline); visit 2, week 1; visit 3, week 4; visit 4, week 12; study exit.

### Patients

A total of 50 DED patients between 18 and 75 years of age were enrolled in the clinical trial after screening. The inclusion criteria were as follows: diagnosed with dry eye according to the criteria of the International Dry Eye Workshop (13), visual acuity > 0.1, and not using any topical eye drugs except artificial tears. The exclusion criteria were as follows: inflammation or infection of lacrimal drainage system, obstruction or stenosis of nasolacrimal duct, conjunctival relaxation, being allergic to the ingredients of hydroxybutyl chitosan such as marine food (since HBC is a derivative of chitin, a high molecular compound purified from shrimp shell), abnormal lid anatomy, active inflammation or infection of cornea and conjunctiva, ocular surgery or trauma within 6 months, having undergone permanent punctal occlusion or absorbable punctal occlusion within 6 months, history of myopia laser surgery, use of contact lens within 1 month, glaucoma, autoimmune diseases, and severe cardiovascular, hepatic, renal or hematopoietic diseases.

### HBC Injection Protocol

The hydroxybutyl chitosan solution is preserved in sterile tubes at 4–8°C. Before injection, drop topical anesthetics to the conjunctival sac to release the unpleasant feeling during the operation. Syringe the upper and lower lacrimal passages in both eyes with saline before injection to ensure the passages are unobstructed. Attach a rinse needle to the tubes containing HBC solution and insert the needle 2–3 mm into the punctum. Inject the HBC solution into the puncta of the upper and lower canaliculi in both eyes until overflow. At the same time, press the dacryocyst sac to avoid downflow of the HBC solution. After injection, press the dacryocyst sac for another 1 min and give antibiotic eye drops to prevent infection. The treatments for all participants were conducted by the same operator.

### Outcome Measures

Outcome measures include safety and changes related to efficacy. All the patients underwent an Ocular Surface Disease Index (OSDI) questionnaire, tear break-up time (TBUT), corneal fluorescence staining (CFS), OCT imaging for tear meniscus height (TMH), and phenol red thread test at Day 0 (baseline) and Weeks 1, 4, and 12. Ocular adverse events (AEs) related to the treatment are recorded.

#### Dry Eye Questionnaire

Ocular Surface Disease Index was used to assess the subjective symptoms at each visit. The questionnaire consisted of the bothersome symptoms, visual function, and environmental triggers subscales. The subjective symptoms were scored on a 5-point scale, with a score of 0 indicating least severe, and a score of 4 indicating most severe. A derived index score of ≤ 100 was calculated for each evaluation based on the total number of questions answered (14).

#### Tear Break-Up Time

TBUT was used to assess the tear film stability. A fluorescein-impregnated strip (Jingming, Tianjin, China) was wetted with saline solution before use. Placed the wetted strip in the lower conjunctival sac and the patient was asked to blink several times. TBUT was defined as the time between the last complete blink and the first black spot appearing in the stained tear film on the cornea. It was measured three times to get the mean TBUT.

#### Corneal Fluorescence Staining

According to the NEI criteria, CFS characteristics in five corneal zones were scored on a 4-point scale as follows: 0 = no staining, 1 ≤ 5 staining points, 2 ≥ 5 staining points but <10 staining points, and 3 ≥ 10 staining points or the appearance of corneal filaments (15).

#### Phenol Red Thread Test

Tear secretion was measured by the phenol red thread test without topical anesthesia. The phenol red thread (Jingming, Tianjin, China) was placed approximately 1/3 of the distance from the lateral canthus of the lower eyelid. The length of the wetted thread was measured as the lacrimal secretion 15 s after placement.

#### Tear Meniscus Height

The lower TMH was measured using an anterior segment OCT system (RTVue-100, Optovue Inc., Freemont, CA, United States) (16). The single-line scanning mode by the anterior segment-wide angle lens was selected (scanning line length, 3 mm; scanning direction, 90°–270°). Patients were instructed to stabilize their heads by an adjustable chin rest and then look straight ahead at an external light-emitting diode target in front of the eye examined. The patients were told to blink normally to evenly distribute the tear film and minimize ocular surface dehydration. Immediately after the patient blinked, scanning started at the 6 o'clock position of the cornea. The participants were asked to hold their blink during the scan. Three consecutive scans were performed during each examination, with a scanning interval of 3–5 s. The TMH was determined from the OCT images with the RTVue-100 image analysis software, which was defined as the straight-line distance between the upper extreme and the lower extreme of the tear boundary line.

### Statistical Analysis

Statistical data were analyzed using SPSS 19.0 (SPSS, Chicago, IL, United States). Results were expressed as means ± SD. Differences were considered to be significant at a level of *P* < 0.05, using *t*-tests or Chi-square tests for inter-group comparisons and repeated measures analysis of variance or non-parametric tests for intra-group comparisons.

## Results

Fifty patients were enrolled in the trial and were randomized into two groups: the HBC group (intracanalicular HBC injection) and the VisiPlug group; 25 patients in each group, respectively. Eight patients dropped out after treatment since they refused follow-up. Twenty patients of the HBC group and 22 patients of the VisiPlug group (total 42 patients) completed the clinical trial (Table 1).

**Table 1:** Characteristics of the study population and the baseline.

	**HBC group**	**VisiPlug group**	***P*-value**
Patients enrolled, *n*	25	25	
Patients completing the study, *n* (%)	20 (80%)	22 (88%)	
Gender, *n* (%)			0.108
Male	4 (20%)	10 (45.5%)	
Female	16 (80%)	12 (54.5%)	
Mean age (years)	44.60 ± 14.82	44.86 ± 13.47	0.952
OSDI	51.21 ± 26.75	40.81 ± 20.59	0.163
Phenol red thread test	3.75 ± 2.17	4.45 ± 2.24	0.308
TBUT	6.47 ± 3.08	6.08 ± 2.61	0.658
Tear meniscus height	184.40 ± 48.83	200.18 ± 41.30	0.263
CFS	0.75	0	0.069

*OSDI, Ocular Surface Disease Index; TBUT, tear break-up time; CFS, corneal fluorescein staining*.

The distribution of age, gender, and baseline DED indexes (OSDI, TBUT, CFS, TMH, and phenol red thread test) between the two treatment groups were all well balanced (Table 1). No significant differences were found. The mean age of all the 42 patients was 44.7 ± 13.8 years. Among them, 28 (66.7%) were women.

### Efficacy

The assessment of efficacy puts emphasis on both the efficacy of intracanalicular HBC injection and the difference between the HBC group and the VisiPlug group in treating DED.

**Figure 2:**
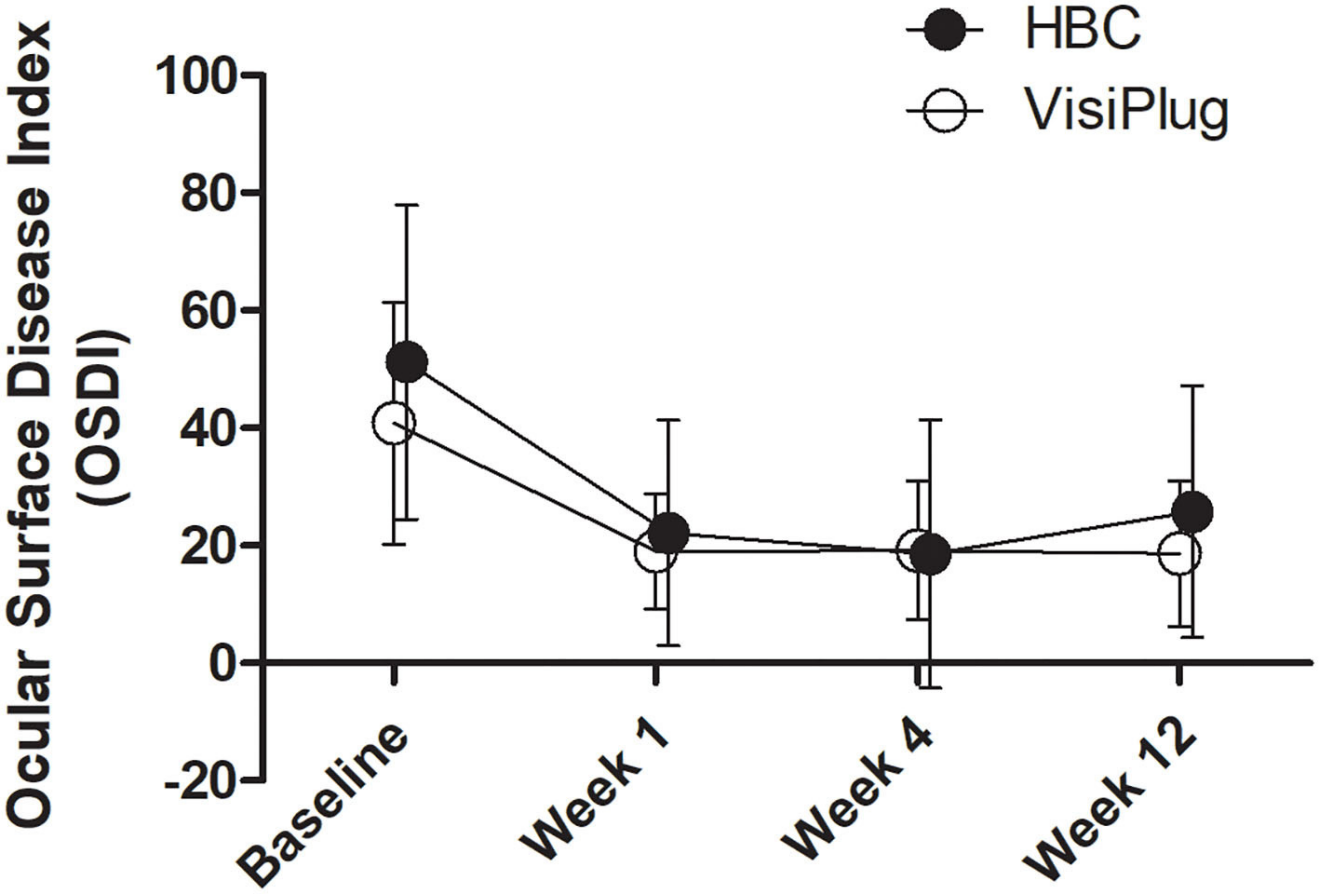
Mean changes of Ocular Surface Disease Index (OSDI) after the treatment in the HBC group and the VisiPlug group. Both groups showed decreased OSDI score compared to baseline (*P* < 0.05) at Weeks 1, 4, and 12. No difference was found between the groups (*P* > 0.05).

The subjective symptoms of DED were relieved in both groups (Figure 2). One week after treatment, the OSDI score was 22.15 ± 19.18 in the HBC group and 19.02 ± 9.72 in the VisiPlug group, which significantly decreased compared to the baseline of each group (HBC: 51.21 ± 26.75; VisiPlug: 40.81 ± 20.59; both *P* < 0.001), indicating an onset effect of as early as 1 week. At Weeks 4 and 12, the OSDI score basically maintained in the VisiPlug group (week 4: 19.19 ± 11.71; week 12: 18.65 ± 12.35; *P* < 0.05 compared to baseline). While in the HBC group, the OSDI score slightly increased at week 12 (week 4: 18.55 ± 22.83; week 12: 25.78 ± 21.43; *P* < 0.05 compared to baseline). No statistical difference of the OSDI score was found between the HBC group and the VisiPlug group at any time (*P* > 0.05). So, it is believed that the HBC injection and the VisiPlug could improve the symptom of DED equally.

**Figure 3:**
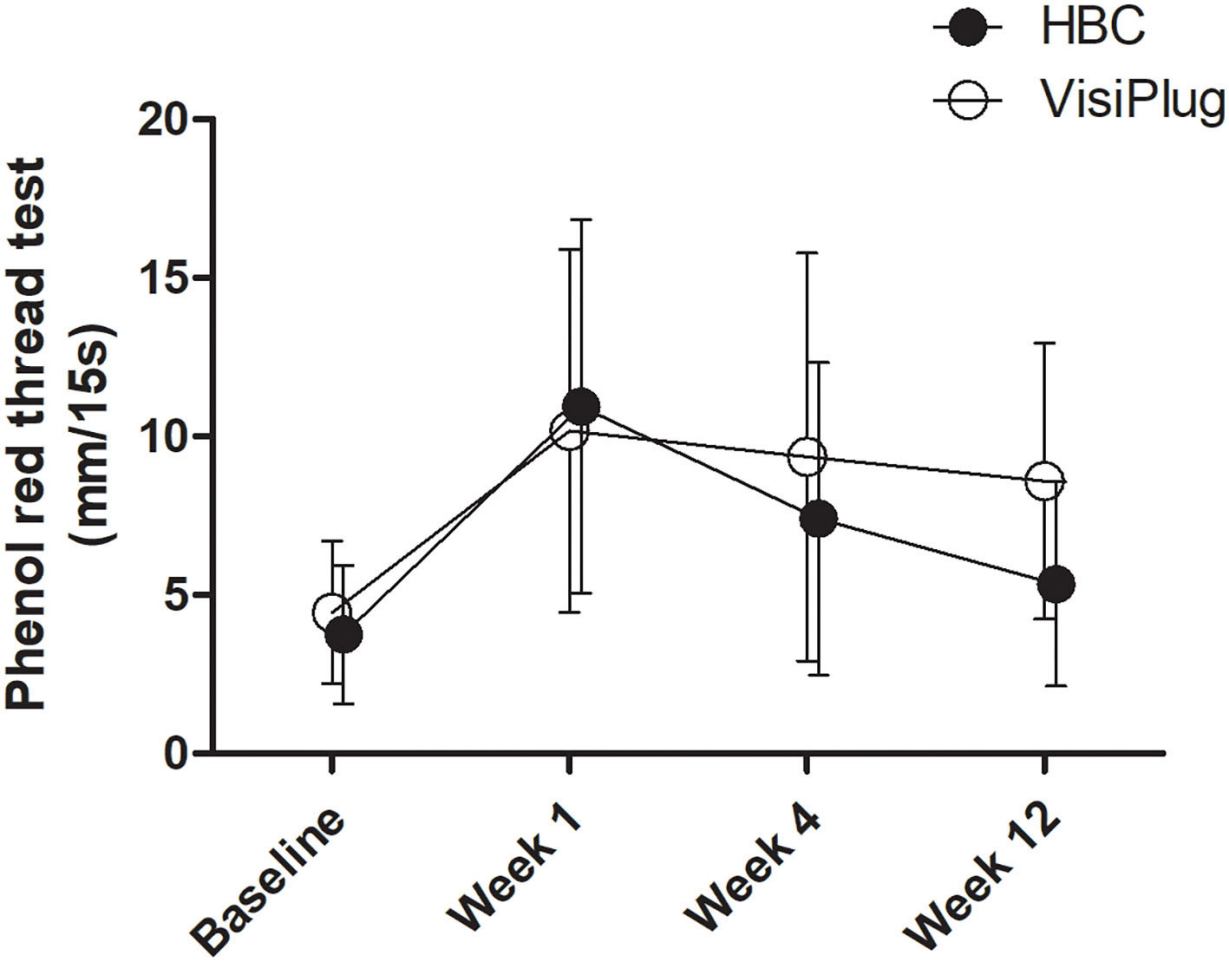
Mean changes of phenol red thread test after the treatment in the HBC group and the VisiPlug group. Both groups showed improvement in phenol red thread test compared to baseline (*P* < 0.05) at Weeks 1, 4, and 12. VisiPlug was better than HBC at Week 12 (*P* = 0.009).

Improvements were also observed in the phenol red thread test after treatment (Figure 3). Generally, phenol red thread test increased greatly at week 1 (HBC: 10.95 ± 5.89 mm, VisiPlug: 10.18 ± 5.72mm) from the baseline (HBC: 3.75 ± 2.17 mm, VisiPlug: 4.45 ± 2.24 mm), and then began to decrease at week 4 (HBC: 7.40 ± 4.93 mm, VisiPlug: 9.36 ± 6.43 mm) and week 12 (HBC: 5.35 ± 3.22 mm, VisiPlug: 8.59 ± 4.35 mm). Notably, there is a significant difference between the two groups at week 12 (*P* = 0.009). The results showed that both groups were effective in improving tear secretion. However, 12 weeks after treatment, the HBC group was not as effective as the VisiPlug group. We suppose that it is caused by the difference in the degradation speed of different absorbable materials.

**Figure 4:**
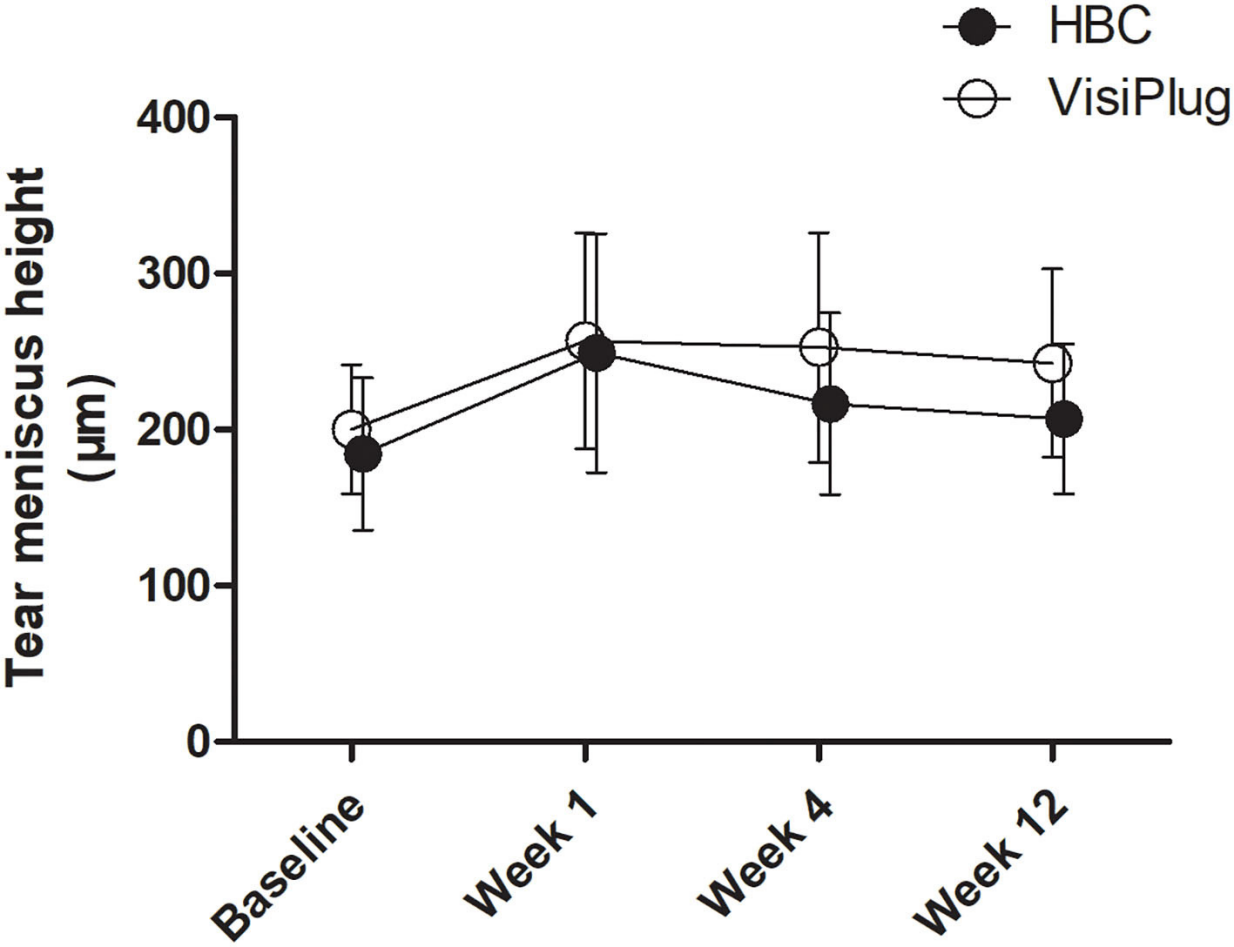
Mean changes of tear meniscus height (TMH) after the treatment in the HBC group and the VisiPlug group. Both groups showed improvement in TMH compared to baseline (*P* < 0.05) at Weeks 1, 4, and 12. VisiPlug was better than HBC at Week 12 (*P* = 0.041).

Both treatment groups showed an improvement in TMH (Figure 4). After treatment, patients in the VisiPlug group had a higher TMH (Week 1: 256.77 ± 69.05 μm; Week 4: 252.68 ± 73.4 μm; Week 12: 242.59 ± 60.30 μm) compared to the baseline (200.18 ± 41.30 μm, all *P* < 0.05). Likewise, TMH in the HBC group increased after HBC injection (Week 1: 249.05 ± 76.54 μm; Week 4: 216.60 ± 58.24 μm; Week 12: 206.9 ± 47.95 μm) compared to the baseline (184.40 ± 48.83 μm, all *P* < 0.05). Similarly, TMH also reflects the tear secretion, so that the VisiPlug treatment was better than the HBC injection in improving TMH at Week 12 (*P* = 0.041).

**Figure 5:**
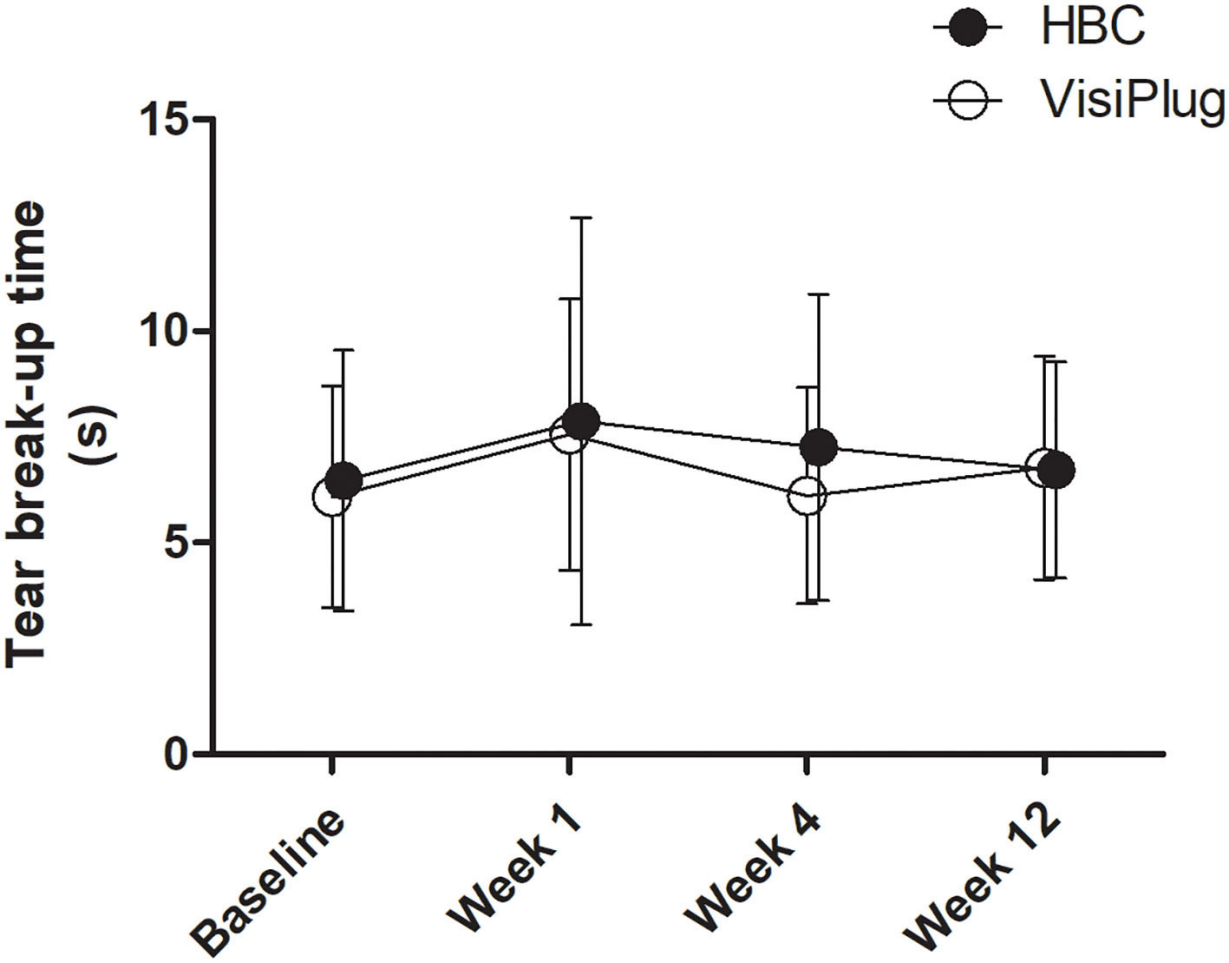
Mean changes of tear break-up time (TBUT) after the treatment in the HBC group and the VisiPlug group. Both groups had little influence on TBUT compared to baseline (*P* > 0.05).

Both groups seemed to have little effect on TBUT (Figure 5). Although some improvement was found at Week 1 (HBC: 7.87 ± 4.81 s; VisiPlug: 7.55 ± 3.21 s) compared to the baseline (HBC: 6.47 ± 3.08 s; VisiPlug: 6.08 ± 2.61 s), there was no statistical difference (HBC: *P* = 0.127; VisiPlug: *P* = 0.060). TBUTs at Weeks 4 and 12 also had no difference with baseline in both groups.

No significant change in CSF was observed in both the inter-group comparisons or the intra-group comparisons, either. It is probably caused by the fact that all the patients enrolled showed little or no CFS score even before the treatment (HBC 0.75; VisiPlug 0).

### Safety

During the clinical trial, no serious AE was reported in the HBC group or the VisiPlug group. The AEs include epiphora, increased secretion, conjunctival congestion, foreign body sensation, eye itching, and blurred vision. All of them were of mild intensity, which relieved automatically without any treatment. They are specifically listed in Table 2.

**Table 2:** Adverse events observed after occlusion treatments.

	**HBC group (*n* = 20)**	**VisiPlug group (*n* = 22)**
Epiphora	3 (15%)	5 (22.7%)
Increased secretion	2 (10%)	3 (13.6%)
Conjunctival congestion	1 (5%)	1 (4.5%)
Foreign body sensation	0 (0%)	2 (9.0%)
Eye itching	1 (5%)	1 (4.5%)
Vision blurred	1 (5%)	0 (0%)

*AE, adverse event*.

*AE is defined as an ocular adverse event occurring after the occlusion treatment*.

## Discussion

A highly comfortable and individualized design of intracanalicular plugs is hard to achieve if they have already been shaped into a certain size before use. Liquid materials can just fit the canaliculus of every person without spatial restriction and are hopeful to occlude canaliculi if they can form a gel after injection *in vivo*. An innovative thermosensitive phase-changing biomaterial, hydroxybutyl chitosan (HBC), makes the idea possible. Research studies showed that HBC can change from a liquid phase to a hydrogel solid phase in a short time. At body temperature (37°C), the phase-changing process will finish in 50 s (11). When it becomes a hydrogel plug, HBC can act as a water barrier (17). These two features make HBC an ideal material for “liquid plug.”

In a previous study, we proposed the feasibility of applying HBC to intracanalicular occlusion and explored its efficacy in a pilot study of eight DED patients (12). To further confirm its efficacy, we enlarged the population to 42 patients and compared the HBC injection to a plug we commonly use nowadays, VisiPlug. In this study, the HBC injection showed similar efficacy as VisiPlug. The HBC injection could improve the symptoms and signs of DED just as the traditional absorbable plugs did during the visit time from Weeks 1 to 12. However, we found that the HBC injection was not as effective as VisiPlug in improving TMH and phenol red thread test at Week 12, which means the therapeutic effect of improving tear secretion may decrease faster in HBC injection as the time goes. Since the duration of the therapeutic effect is related to the degradation speed of the absorbable materials (18), we believe that the phenomenon is due to a faster degradation speed of HBC injection than VisiPlug. As the material degrades, more volume of tears pass through the lacrimal drainage system. Based on the clinical data, we suppose the effect of HBC will last for at least 4 weeks. To maintain the efficacy, DED patients may need a relatively frequent HBC injection therapy.

Both the HBC injection and the VisiPlug are methods that treat DED through lacrimal drainage occlusion. So, HBC injection had similar AEs as traditional absorbable intracanalicular plugs (19–21) in this study, including epiphora, increased secretion, conjunctival congestion, foreign body sensation, eye itching, and blurred vision. Besides these, there was no other special peculiar adverse event or complication after HBC injection in this study. We also did not find any difference in the rate or severity of AEs between them, except for the foreign body sensation (HBC: 0%; VisiPlug: 9%). We know that after injection, HBC will turn into a gel-like plug of which the size is just the same as the canaliculi of the patient, so fewer foreign body sensation events may owe to the special characteristics of HBC. The “liquid plug” design will bring more comfort to DED patients after occlusion therapy. In the previous study, we have already confirmed the biosafety of HBC to the ocular surface and an acceptable, transient inflammation reaction to the canalicular (12). We believe the safety of HBC injection is convincing.

The most important advantage of HBC injection occlusion over other traditional plugs is its individualized and flexible feature. Operators do not need to consider the problems of size or accidents such as spontaneous plug extrusion. Another potential advantage we believe is that HBC injection is less likely to cause infection: HBC can exhibit antibacterial activities just like chitosan (22). Furthermore, the phase transformation will lead to a retraction in volume, which can avoid complete obstruction of canaliculi (23). A more extensive application is needed to support this opinion.

One limitation of our findings is that we lack an appropriate method to monitor the HBC plug after injection. MR is used previously to image the plug (12) (water in the gel can be imaged in the T2 sequence) but may be not suitable for long-term observation since the water content decreases as time goes on. Future explorations should emphasize these problems.

## Conclusions

The intracanalicular HBC injection was able to relieve both symptoms and signs of dry eye disease with great safety. No significant difference was found between the HBC injection and VisiPlug either in the efficacy of symptom improvement or in the safety. Taking advantage of the thermosensitive and dissolving properties of HBC, the *in situ* injection of HBC solution into the lacrimal drainage system proves to be a promising, individualized occlusion method to treat dry eye disease.

## Data Availability Statement

The original contributions presented in the study are included in the article/supplementary material, further inquiries can be directed to the corresponding author/s.

## Ethics Statement

The studies involving human participants were reviewed and approved by the Ethical Committee of Eye & ENT Hospital of Fudan University. The patients/participants provided their written informed consent to participate in this study.

## Author Contributions

TL and LG designed the study. TL, YL, and LG performed recruitment, treatment, and follow-up for dry eye patients. TL and WW analyzed the data. TL, WW, and LG wrote and revised the manuscript. All authors approved the final manuscript.

## Funding

This study was financially supported by Shanghai Sailing Program (Grant No. 19YF1405800 to LT), the National Natural Science Foundation of China (Grant No. 82000855 to LT), and the Shanghai Rising Stars of Medical Talents Youth Development Program (Youth Medical Talents Specialist Program to LT).

## Conflict of Interest

The authors declare that the research was conducted in the absence of any commercial or financial relationships that could be construed as a potential conflict of interest.

## Publisher's Note

All claims expressed in this article are solely those of the authors and do not necessarily represent those of their affiliated organizations, or those of the publisher, the editors and the reviewers. Any product that may be evaluated in this article, or claim that may be made by its manufacturer, is not guaranteed or endorsed by the publisher.
